# Using Patient-Reported Outcomes to Describe the Patient Experience on Phase I Clinical Trials

**DOI:** 10.1093/jncics/pkaa067

**Published:** 2020-08-14

**Authors:** Ramy Sedhom, Betty Ferrell, Nora Ruel, Marianna Koczywas, Vincent Chung, Thomas J Smith

**Affiliations:** 1 Johns Hopkins Sidney Kimmel Comprehensive Cancer Institute, Baltimore, MD, USA; 2 City of Hope National Medical Center, Duarte, CA, USA

## Abstract

**Background:**

Symptoms are common among patients enrolled in phase I trials. We assessed the validity of Patient-Reported Outcomes version of the Common Terminology Criteria for Adverse Events (PRO-CTCAE) items in relation to previously validated assessments of quality of life and psychological distress. We used data from a randomized trial testing a palliative care support intervention for patients enrolled on phase I trials.

**Methods:**

Patients (n = 479) were accrued to the parent study prior to initiating a phase I clinical trial with data collected at baseline, 4, and 12 weeks. We determined the correlation of PRO-CTCAE with distress level, Functional Assessment of Cancer Therapy - General (FACT-G) total, and subscale domain scores.

**Results:**

Patients were predominantly female (56.8%) and older than age 60 years, and 30.7% were from minority populations. The correlation coefficient for distress level for all PRO-CTCAE items was small to moderate (Pearson *r *=* *0.33-0.46). Pearson correlation coefficient for FACT-G total was moderate (*r* = -0.45 to -0.69). Stronger associations were noted for mood items of the PRO-CTCAE only (with distress level, *r *=* *0.55-0.6; with FACT-G, *r* = -0.54 to -0.6). PRO-CTCAE symptom interference scores had the strongest correlation with distress level (Pearson *r *=* *0.46) and FACT-G total (Pearson *r* = -0.69). Correlations between PRO-CTCAE items and corresponding FACT-G (total and subscales) and distress levels reached statistical significance for all items (*P* <.001).

**Conclusion:**

Evidence demonstrates validity of PRO-CTCAE in a heterogeneous US sample of patients undergoing cancer treatment on phase I trials, with small to moderate correlations with distress level for all PRO-CTCAE items and moderate correlations with quality of life as measured by FACT-G total.

Standard adverse event (AE) reporting in phase I clinical trials has historically not engaged patients to self-report symptoms, leading to potential underestimation of harms, both at baseline and over the course of a trial (1–10). There is a growing body of evidence supporting the use of patient-reported outcomes (PROs) in oncology clinical trials (11–22). The National Cancer Institute’s (NCI) Patient-Reported Outcomes version of the Common Terminology Criteria for Adverse Events (PRO-CTCAE) was developed to allow patients to self-report symptomatic AEs and improve the quality of symptomatic AE detection (23). Attention to the patient experience is essential for optimal care, especially as health-related quality of life is becoming an integral part of cancer clinical trials (16,24–26).

Industry sponsors are now beginning to implement PRO-CTCAE across the continuum of trials including early phase, phase III, and postmarketing studies. The US Food and Drug Administration has also encouraged adoption of this tool in oncology trials (27,28). Historically, the most common PRO strategy for oncology has been to assess the broad multidomain concept of health-related quality of life (28–30). These existing measures have strengths, including familiarity with their use among the cancer therapeutic development community, but they often ask questions less relevant to the trial context and/or miss the assessment of important symptoms. This limitation is especially important in the era of novel cancer therapeutics, where adverse events can differ from traditional cytotoxic chemotherapy because of novel mechanisms of action, continuous oral administration of therapy, and more prolonged duration of treatment.

Although phase I studies are primarily focused on treatment safety and feasibility, it remains important to both quantify mild or moderate adverse events and assess their impact on patient function and well-being. This concept of “treatment tolerability” becomes increasingly important in an era where patients are living longer and cancer is increasingly managed as a chronic disease (31). Few investigators conducting phase I oncology trials have explored whether PRO-CTCAE is correlated with important patient measures, such as distress and quality of life.

The PRO-CTCAE is an item library that includes individual patient questions representing 78 unique symptomatic AEs (23). Items are explained in patient-friendly language and have undergone rigorous psychometric development and validation (32,33). The PRO-CTCAE includes up to 3 discrete questions for each AE, separately representing the frequency (F), severity (S), and/or interference (I) of each event. Items are available from the NCI at http://healthcaredelivery.cancer.gov/pro-ctcae.

It is essential to establish that PRO-CTCAE accurately and reliably captures the underlying experience it is intended to measure. To accomplish this, we performed a secondary analysis of all patients who enrolled on a randomized trial investigating a palliative care intervention for cancer patients enrolled on phase I trials at 2 institutions. We evaluated the measurement properties of several items in the PRO-CTCAE library and correlated these items with the Distress Thermometer and FACT-G total and subscale scores. These anchors were primary endpoints of the main parent trial and were chosen given published studies associating patient quality of life and distress with physical function and symptom burden (34–36).

## Methods

### Design

This secondary analysis of patient-reported outcome data was derived from a randomized clinical trial funded by the NCI to test integration of palliative care for patients beginning a phase 1 trial. In the parent study, patients with solid tumors (n = 479) were accrued from 2 NCI Comprehensive Cancer Centers with baseline data collected prior to the initial phase I treatment. Patients were randomly assigned to usual care or the palliative care intervention. Procedures for patients in the palliative care intervention group included a care plan created by the study nurse based on data from the baseline evaluation and a discussion of the patient in an interdisciplinary meeting of the study investigators, nurses, a chaplain, and a social worker, and the patient received 2 teaching sessions by the research nurse using standardized teaching materials addressing symptom and quality-of-life (QOL) concerns. Follow-up evaluation occurred at 4 and 12 weeks.

The primary outcome of the parent study was to test the effects of a palliative care intervention on patients’ quality of life, psychological distress, and satisfaction with oncology care and communication. To qualify for the study, patients were required to be 21 years of age or older, fluent in English, without cognitive impairment, diagnosed with a solid tumor, and initiating treatment on a phase I clinical trial. Exclusion criteria included cognitive impairment and hematologic malignancy. The trial was approved by institutional review boards at each site and registered with ClinicalTrials.gov ID NCT01612598. Written informed consent was provided by each participant.

### Questionnaire

The previously developed PRO-CTCAE item library consists of 78 symptomatic AEs represented by 124 distinct items (23). When planning for this study, we met with stakeholders from the NCI and selected a pool of 45 items that were deemed relevant to the study population (see Table 1). PRO-CTCAE items were completed by phase I trial participants prior to clinic appointments. Participants were required to answer questions without assistance but could request technical assistance from study staff.

**Table 1.** List of PRO-CTCAE items used in the aggregate scoring^a^

No.	PRO-CTCAE	Category	S (n = 39)	I (n = 21)	F (n = 10)	P (n = 2)
1	Problems with concentration	Attention/Memory	X	X	—	—
2	Problems with memory	Attention/Memory	X	X	—	—
3	Arm or leg swelling	Cardio/Circulatory	X	X	—	—
4	Pounding or racing heartbeat (palpitations)	Cardio/Circulatory	X	—	X	—
5	Tremors	Cardio/Circulatory	X	—	X	—
6	Acne and pimples	Cutaneous	X	—	—	—
7	Hair loss	Cutaneous	—	—	—	X
8	Hand and foot syndrome	Cutaneous	X	—	—	—
9	Problems with nails	Cutaneous	X	—	—	—
10	Skin burns from radiation	Cutaneous	X	—	—	—
11	Skin problems	Cutaneous	X	X	—	—
12	Bloating of abdomen	Gastrointestinal	X	—	X	—
13	Constipation	Gastrointestinal	X	—	—	—
14	Decreased appetite	Gastrointestinal	X	X	—	—
15	Heartburn	Gastrointestinal	X	—	—	—
16	Hiccups	Gastrointestinal	X	—	X	—
17	Loose stools (diarrhea)	Gastrointestinal	—	X	X	—
18	Nausea	Gastrointestinal	X	—	—	—
19	Problems tasting food or drink	Gastrointestinal	X	—	—	—
20	Vomiting	Gastrointestinal	X	—	—	—
21	Urge to urinate	Gynecologic/Urinary	—	X	X	—
22	Frequent urination	Gynecologic/Urinary	—	X	X	
23	Bruise easily	Miscellaneous	—	—	—	X
24	Hot flashes	Miscellaneous	X	—	—	—
25	Shivering	Miscellaneous	X	—	—	—
26	Excessive sweating	Miscellaneous	X	—	X	—
27	Anxiety	Mood	X	X	X	—
28	Depression	Mood	X	X	—	—
29	Dizziness	Neurological	X	X	—	—
30	Numbness in hands and feet	Neurological	X	X	—	—
31	Difficulty swallowing	Oral	X	—	—	—
32	Dry mouth	Oral	X	—	—	—
33	Mouth sores	Oral	X	X	—	—
34	Skin cracking at mouth	Oral	X	—	—	—
35	Headache	Pain	X	X	—	—
36	Pain	Pain	X	X	—	—
37	Problems with breathing	Respiratory	X	X	—	—
38	Cough	Respiratory	X	X	—	—
39	Shortness of breath	Respiratory	X	X	—	—
40	Decreased sexual interest	Sexual	X	—	—	—
41	Problems with ejaculation	Sexual	—	—	X	—
42	Fatigue	Sleep	X	X	—	—
43	Insomnia	Sleep	X	X	—	—
44	Blurry vision	Visual/Perceptual	X	X	—	—
45	Ringing in ears	Visual/Perceptual	X	—	—	—

a Em dashes indicate items not used for this measure. F = frequency; I = interference; P = problem/presence; PRO-CTCAE = Patient-Reported Outcomes version of the Common Terminology Criteria for Adverse Events; S = severity.

### Quantitative Data and Anchors

A demographic data tool and 2 well-validated, patient-reported psychosocial measures were used as comparators in the instrument validation. These PRO anchors were administered to participants prospectively and selected based on literature review and expert consensus.

### Psychological Distress Scale

The Psychological Distress Scale is a single item asking patients to rate their distress on a scale of 0  (none) to 10  ( extreme distress) (37). A mark of 5 or above indicates a need for intervention.

### FACT-G

The FACT-G is a well-established validated QOL scale consisting of 27 items rated on a 0-4 scale. The tool includes subscales of physical well-being, social/family well-being, emotional well-being (EWB), and functional well-being, and overall QOL. All of the FACT-G items have a 5-point scale from 0 to 4 for responses ranging from “not at all” to “very much.” The highest possible score for the EWB subscale is 24, and 28 for the other 3 subscales. Thus, the total FACT-G score can range from 0 to 108, with higher scores indicating better QOL. Subscale scores can be prorated for defined missing data (38).

### Statistical Analysis

This study was designed as a 2-group experiment, powered to detect statistically significant group differences in QOL and related metrics in the intervention and control cohorts over time. As the patient-reported outcome components were part of the secondary endpoints in our study, the parent study was not powered to analyze the data specific to this tool.

Aggregate scores using PRO-CTCAE were calculated to explore the effect of overall symptom frequency (10 items), symptom severity (39 items), and symptom interference (21 items), by calculating the total of all scored items classified within each of those attributes. Dueck et al. implemented an intricate scoring system based on permutations of responses to the 45 questions (39). Our goal was to take a simplified approach, which measured the overall load of severity (S), interference (I), and frequency (F) of patient-reported outcomes (the 2 items related to presence of symptoms were not included in these calculations). The list of items used to compose each of these overall scores is found in Table 1. We used these metrics to identify associations between this and other validated tools.

To assess convergent validity, baseline scores were used to compute Pearson correlations between each PRO-CTCAE attribution group (F, S, I), and Functional Assessment of Cancer Therapy: General (FACT-G) Health Related Quality of Life (HRQOL) summary, subscales, and distress level. Corresponding calculations using scores at subsequent time points were also considered. Correlation values less than 0.3 were considered negligible, 0.3-0.5 small, and 0.5-0.7 as moderate in our comparisons (40). When applicable, *P* values were provided to indicate the probability of seeing a Pearson correlation coefficient greater than the observed values, under the null hypothesis that the coefficient was equal to 0, thus using a 2-sided test. The cut point used for statistical significance was .001, because a number of coefficients were tested.

## Results

### Demographic Data

Characteristics of the participants are presented in Table 2. Patients were predominantly female (56.8%) and older than age 60 years (55.5%), and 30.7% were from ethnic minority populations. Median number of comorbidities was 1, with a majority of patients reporting 1 (25.9%), 2 (22.8%), or 3 (15.9%) comorbidities. Religious affiliations included 16.5% no affiliation, 29.4% Catholic, 5.8% Jewish, 38.8% Protestant, and 9.0% other religions.

**Table 2.** Patient demographics^a^

Patient demographic variables	All patients
	(n = 479)
Treatment arm, No. (%)	
Experimental	240 (50.1)
Control	239 (49.9)
Age, median (IQR), y	62 (53-69)
Age, No. (%), y	
<50	85 (17.8)
50-54	51 (10.6)
55-59	77 (16.1)
60-64	71 (14.8)
65-69	89 (18.6)
70-74	57 (11.9)
75-79	36 (7.5)
≥80	13 (2.7)
Gender, No. (%)	
Female	272 (56.8)
Male	207 (43.2)
Race/Ethnicity, No. (%)	
African American	34 (7.1)
Asian	46 (9.6)
Caucasian	332 (69.3)
Hispanic Latino	43 (9.0)
Native Hawaiian	6 (1.3)
Mixed Race	12 (2.5)
Other	6 (1.3)
Religion, No. (%)	
None	79 (16.5)
Protestant	186 (38.8)
Catholic	141 (29.4)
Jewish	28 (5.8)
Other	43 (9.0)
No Response	2 (0.4)
Type of cancer, No. (%)	
Bladder/Urinary	17 (3.5)
Breast	38 (7.9)
Cervical/Uterine	17 (3.5)
Colon	85 (17.7)
Other gastrointestinal	27 (5.6)
Lung	74 (15.4)
Melanoma	11 (2.3)
Oral	11 (2.3)
Ovarian	42 (8.8)
Pancreatic	42 (8.8)
Prostate	21 (4.4)
Rectal	28 (5.8)
Renal	23 (4.8)
Sarcoma	13 (2.7)
Other	30 (6.3)
No. of comorbidities, median (IQR)	1 (1-3)

a IQR = interquartile range.

### Quantitative Data: PRO-CTCAE Scores and Correlation With Other Validated Tools

PRO symptom frequency, interference, severity, and problem and/or presence (P) are scored from 0 (not at all, no problem, or none) to 4 (all the time, big problem, a lot). Frequencies of symptoms at baseline and follow-up (unadjusted and adjusted rates) are reported in Table 3, with number (percentage) of patients who reported any symptoms with grade 0 or higher and number reporting symptoms with grade 3 or higher. Symptom levels reported at 4- and 12-week follow-up were combined to reflect the highest level across both time points for each symptom. Unadjusted scores reflect the worst or highest level of each symptom reported during both follow time points, without consideration of symptoms at baseline. Adjusted scores were obtained using the baseline grade subtraction method for the patient report (24), which takes into account the level of the PRO reported at baseline, with intent to identify the number of patients whose symptom worsened at either point in follow-up. Therefore, adjusted scores represent the subset of patients whose symptom for a PRO item was worse in follow-up (week 4 or 12) than at baseline, discounting the individuals who may have reported symptoms that were the same or improved from the baseline observation. This was deemed important to capture overall symptom burden and how symptoms changed over time given our interest to correlate with both patient distress and quality of life. Almost all participants reported the presence of at least 1 symptom (ie, a score of >0) at baseline.

**Table 3.** Frequency (%) of PRO-CTCAE items at baseline, with maximum unadjusted and adjusted scores in follow-up (week 4 and week 12), in decreasing order of frequency at baseline within category^a^

PRO-CTCAE item	Frequency (%)					
	Baseline		Unadjusted (max level recorded W4/W12)		Adjusted (max level recorded W4/W12)	
	(n = 479)		(n = 426)		(n = 426)	
Attention/memory	Score > 0	Score ≥ 3	Score > 0	Score ≥ 3	Score > 0	Score ≥ 3
Problems with memory (S)	217(45.3)	9 (1.9)	262 (61.5)	10 (2.3)	81 (19.0)	6 (1.4)
Problems with concentration (S)	204 (42.6)	11 (2.3)	242 (56.8)	10 (2.3)	82 (19.2)	8 (1.9)
Problems with memory (I)	188 (39.2)	8 (1.7)	238 (55.9)	9 (2.1)	85 (20.0)	8 (1.9)
Problems with concentration (I)	186 (38.8)	15 (3.1)	226 (53.1)	11 (2.6)	89 (20.9)	9 (2.1)
Cardiovascular/Circulatory						
Palpitations (F)	103 (21.5)	2 (0.4)	138 (32.4)	4 (0.9)	71 (16.7)	2 (0.5)
Palpitations (S)	86 (18.0)	3 (0.6)	131 (30.8)	2 (0.5)	77 (18.1)	0 (0.0)
Arm or leg swelling (S)	71 (14.8)	13 (2.7)	113 (26.5)	22 (5.2)	65 (15.3)	16 (3.8)
Arm or leg swelling (I)	49 (10.2)	12 (2.5)	87 (20.4)	21 (4.9)	57 (13.4)	15 (3.5)
Tremors (F)	29 (6.1)	7 (1.5)	42 (9.9)	8 (1.9)	26 (6.1)	5 (1.2)
Tremors (S)	26 (5.4)	4 (0.8)	37 (8.7)	5 (1.2)	23 (5.4)	2 (0.5)
Cutaneous						
Hair loss (P)	155 (32.4)	64 (13.4)	168 (39.4)	74 (17.4)	74 (17.4)	37 (8.7)
Skin problems (S)	108 (22.5)	16 (3.3)	174 (40.8)	16 (3.8)	95 (22.3)	11 (2.6)
Problems with nails (S)	85 (17.7)	8 (1.7)	95 (22.3)	7 (1.6)	40 (9.4)	5 (1.2)
Skin problems (I)	62 (12.9)	13 (2.7)	111 (26.1)	10 (2.3)	77 (18.1)	6 (1.4)
Problems with nails (I)	30 (6.3)	1 (0.2)	45 (10.6)	3 (0.7)	31 (7.3)	3 (0.7)
Hand and foot syndrome (S)	28 (5.8)	3 (0.6)	43 (10.1)	2 (0.5)	28 (6.6)	2 (0.5)
Acne and pimples (S)	26 (5.4)	7 (1.5)	53 (12.4)	4 (0.9)	35 (8.2)	2 (0.5)
Skin burns from radiation (S)	23 (4.8)	2 (0.4)	28 (6.6)	3 (0.7)	11 (2.6)	1 (0.2)
Gynecologic/Urinary						
Frequent urination (F)	191 (39.9)	43 (9.0)	223 (52.3)	52 (12.2)	89 (20.9)	25 (5.9)
Urge to urinate (F)	156 (32.6)	22 (4.6)	197 (46.2)	32 (7.5)	94 (22.1)	17 (4.0)
Frequent urination (I)	125 (26.1)	16 (3.3)	168 (39.4)	33 (7.7)	83 (19.5)	22 (5.2)
Urge to urinate (I)	105 (21.9)	14 (2.9)	155 (36.4)	24 (5.6)	90 (21.1)	14 (3.3)
Gastrointestinal						
Decreased appetite (S)	225 (47.0)	30 (6.3)	278 (65.3)	53 (12.4)	120 (28.2)	39 (9.2)
Constipation (S)	220 (45.9)	15 (3.1)	218 (51.2)	21 (4.9)	83 (19.5)	18 (4.2)
Decreased appetite (I)	181 (37.8)	30 (6.3)	238 (55.9)	52 (12.2)	114 (26.8)	37 (8.7)
Bloating of abdomen (F)	179 (37.4)	26 (5.4)	215 (50.5)	41 (9.6)	98 (23.0)	29 (6.8)
Bloating of abdomen (S)	170 (35.5)	16 (3.3)	206 (48.4)	28 (6.6)	88 (20.7)	23 (5.4)
Diarrhea (F)	156 (32.6)	25 (5.2)	203 (47.7)	29 (6.8)	108 (25.4)	24 (5.6)
Heartburn (S)	144 (30.1)	7 (1.5)	160 (37.6)	3 (0.7)	59 (13.8)	2 (0.5)
Nausea (S)	134 (28.0)	11 (2.3)	191 (44.8)	20 (4.7)	109 (25.6)	17 (4.0)
Problems tasting food (S)	105 (21.9)	10 (2.1)	150 (35.2)	23 (5.4)	86 (20.2)	17 (4.0)
Hiccups (F)	91 (19.0)	5 (1.0)	99 (23.2)	6 (1.4)	46 (10.8)	3 (0.7)
Hiccups (S)	80 (16.7)	1 (0.2)	88 (20.7)	1 (0.2)	40 (9.4)	1 (0.2)
Vomiting (S)	62 (12.9)	8 (1.7)	97 (22.8)	14 (3.3)	65 (15.3)	13 (3.1)
Miscellaneous						
Bruise easily (P)^b^	106 (22.1)	0 (0)	112 (26.3)	0 (0.0)	31 (7.3)	0 (0.0)
Excessive sweating (F)	93 (19.4)	5 (1.0)	125 (29.3)	3 (0.7)	66 (15.5)	2 (0.5)
Excessive sweating (S)	86 (18.0)	4 (0.8)	116 (27.2)	4 (0.9)	67 (15.7)	3 (0.7)
Hot flashes (S)	61 (12.7)	8 (1.7)	72 (16.9)	10 (2.3)	35 (8.2)	5 (1.2)
Shivering (S)	37 (7.7)	6 (1.3)	72 (16.9)	5 (1.2)	52 (12.2)	4 (0.9)
Mood						
Anxiety (F)	354 (73.9)	52 (10.9)	370 (86.9)	34 (8.0)	100 (23.5)	16 (3.8)
Anxiety (S)	349 (72.9)	34 (7.1)	365 (85.7)	19 (4.5)	93 (21.8)	9 (2.1)
Anxiety (I)	248 (51.8)	27 (5.6)	309 (72.5)	22 (5.2)	124 (29.1)	14 (3.3)
Depression (S)	238 (49.7)	14 (2.9)	295 (69.2)	16 (3.8)	111 (26.1)	8 (1.9)
Depression (I)	194 (40.5)	9 (1.9)	251 (58.9)	15 (3.5)	124 (29.1)	12 (2.8)
Neurological						
Numbness in hands and feet (S)	202 (42.2)	15 (3.1)	233 (54.7)	15 (3.5)	76 (17.8)	7 (1.6)
Numbness in hands and feet (I)	155 (32.4)	13 (2.7)	194 (45.5)	13 (3.1)	81 (19.0)	6 (1.4)
Dizziness (S)	107 (22.3)	2 (0.4)	138 (32.4)	4 (0.9)	69 (16.2)	4 (0.9)
Dizziness (I)	84 (17.5)	1 (0.2)	116 (27.2)	5 (1.2)	64 (15.0)	5 (1.2)
Oral						
Dry mouth (S)	200 (41.8)	11 (2.3)	258 (60.6)	17 (4.0)	111 (26.1)	13 (3.1)
Difficulty swallowing (S)	64 (13.4)	2 (0.4)	94 (22.1)	4 (0.9)	43 (10.1)	4 (0.9)
Mouth sores (S)	33 (6.9)	11 (2.3)	65 (15.3)	6 (1.4)	48 (11.3)	4 (0.9)
Mouth sores (I)	23 (4.8)	9 (1.9)	49 (11.5)	7 (1.6)	39 (9.2)	6 (1.4)
Skin cracking at mouth (S)	21 (4.4)	4 (0.8)	39 (9.2)	2 (0.5)	28 (6.6)	2 (0.5)
Pain						
Pain (S)	295 (61.6)	55 (11.5)	336 (78.9)	51 (12.0)	101 (23.7)	27 (6.3)
Pain (I)	258 (53.9)	47 (9.8)	306 (71.8)	44 (10.3)	116 (27.2)	30 (7.0)
Headache (S)	118 (24.6)	8 (1.7)	123 (28.9)	9 (2.1)	52 (12.2)	8 (1.9)
Headache (I)	74 (15.4)	6 (1.3)	92 (21.6)	6 (1.4)	54 (12.7)	5 (1.2)
Respiratory						
Shortness of breath (S)	178 (37.2)	11 (2.3)	227 (53.3)	23 (5.4)	95 (22.3)	19 (4.5)
Shortness of breath (I)	152 (31.7)	17 (3.5)	215 (50.5)	34 (8.0)	113 (26.5)	27 (6.3)
Problems breathing (S)	149 (31.1)	7 (1.5)	206 (48.4)	15 (3.5)	98 (23.0)	11 (2.6)
Cough (S)	139 (29.0)	10 (2.1)	189 (44.4)	10 (2.3)	83 (19.5)	7 (1.6)
Problems breathing (I)	130 (27.1)	4 (0.8)	188 (44.1)	15 (3.5)	97 (22.8)	14 (3.3)
Cough (I)	84 (17.5)	6 (1.3)	136 (31.9)	8 (1.9)	76 (17.8)	5 (1.2)
Sexual						
Decreased sexual interest (S)	178 (37.2)	49 (10.2)	190 (44.6)	57 (13.4)	87 (20.4)	35 (8.2)
Problem with ejaculation (F)	42 (8.8)	10 (2.1)	38 (8.9)	4 (0.9)	20 (4.7)	2 (0.5)
Sleep						
Fatigue and lack of energy (S)	352 (73.5)	90 (18.8)	398 (93.4)	114 (26.8)	151 (35.4)	69 (16.2)
Fatigue and lack of energy (I)	322 (67.2)	102 (21.3)	388 (91.1)	135 (31.7)	169 (39.7)	82 (19.2)
Insomnia (S)	241 (50.3)	23 (4.8)	256 (60.1)	20 (4.7)	85 (20.0)	17 (4.0)
Insomnia (I)	204 (42.6)	20 (4.2)	213 (50.0)	16 (3.8)	81 (19.0)	13 (3.1)
Visual						
Blurry vision (S)	72 (15.0)	5 (1.0)	98 (23.0)	4 (0.9)	46 (10.8)	3 (0.7)
Blurry vision (I)	67 (14.0)	6 (1.3)	92 (21.6)	5 (1.2)	48 (11.3)	5 (1.2)
Ringing in ears (S)	48 (10.0)	8 (1.7)	59 (13.8)	6 (1.4)	28 (6.6)	2 (0.5)

a Unadjusted adverse events (AEs) in follow-up are highest AE reported in week 4 (W4) or week 12 (W12); adjusted AEs in follow-up are highest AE reported in W4 or W12 counted only if level reported higher than at baseline; items scored from 0 (not at all, no problem, none) to 4 (all the time, big problem, a lot). F = frequency; I = interference; P = problem, presence; PRO-CTCAE = Patient-Reported Outcomes version of the Common Terminology Criteria for Adverse Events; S = severity.

b “Bruise easily” is recorded as either present or absent (0 = absent, 1 = present).

At baseline, patients reported frequent problems with memory (S, 45.3%), concentration (S, 42.6%), appetite (S, 47.0%), constipation (S, 45.9%), anxiety (F, 73.9%), depression (S, 49.7%), numbness in hands and feet (S, 42.2%), dry mouth (I, 41.8%), pain (S, 61.6%), fatigue (S, 73.5%), and insomnia (S, 50.3%). Symptoms at baseline that were scored 3 or higher by more than 10% of patients included hair loss (P, 13.4%), anxiety (F, 10.9%), pain (S, 11.5%), sexual interest (S, 10.2%), and fatigue (I, 21.3%).

**Figure 1.**
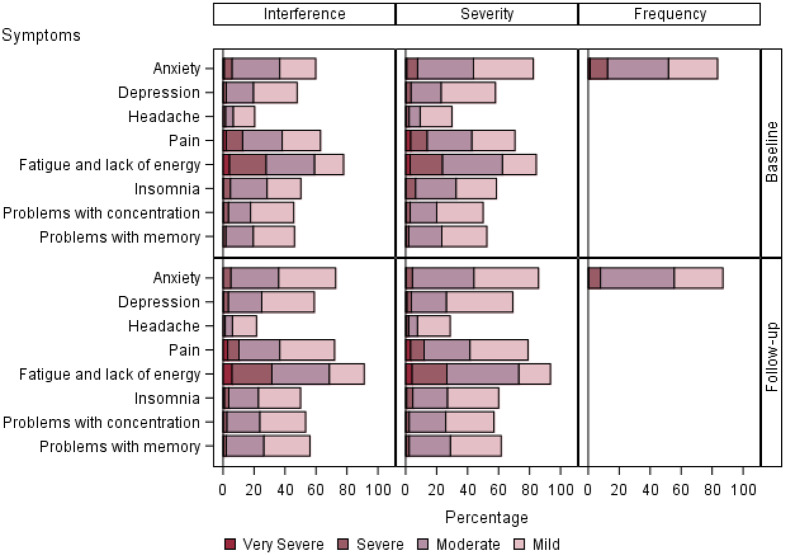
PRO-CTCAE symptoms rated in the categories of mood, pain, sleep, and attention. Bar chart panels highlight symptom attribute and level over time. Bar chart panels break down interference, severity, and frequency attributes of symptoms by level. Responses are detailed with regard to percentage of time each symptom is seen at a given level. PRO-CTCAE = Patient-Reported Outcomes version of the Common Terminology Criteria for Adverse Events.

**Figure 2.**
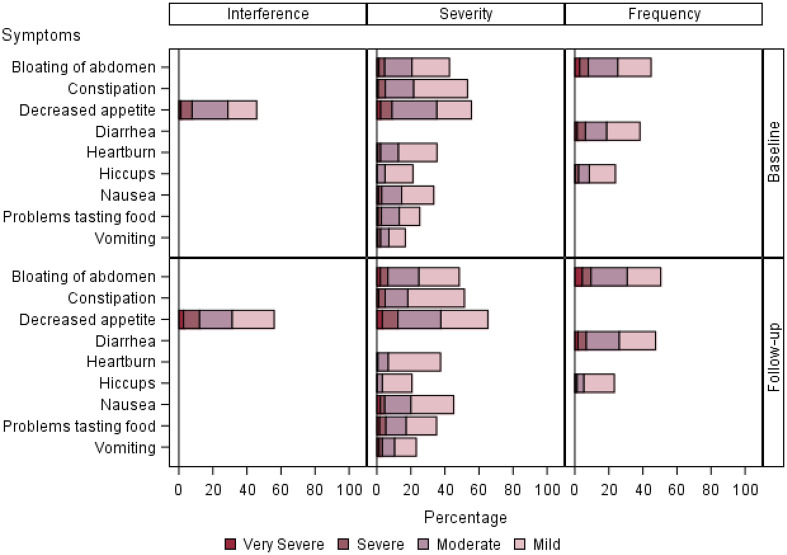
PRO-CTCAE symptoms rated in the gastrointestinal category. Bar chart panels highlight interference, severity, and frequency symptom attribute and level over time. Responses are detailed with regard to percentage of time each symptom is seen at a given level. Gastrointestinal symptoms consisted of some of the symptoms that were reported with higher severities. Appetite was one of the most common issues reported, which increased in severity at follow-up. PRO-CTCAE = Patient-Reported Outcomes version of the Common Terminology Criteria for Adverse Events.

**Figure 3.**
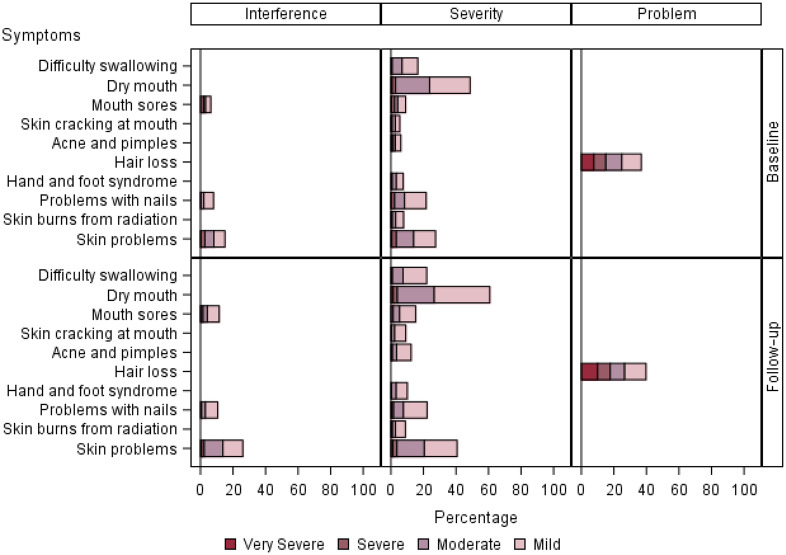
PRO-CTCAE symptoms rated in the categories cutaneous and oral. Bar chart panels highlight symptom attribute and level over time. Bar chart panels break down interference, severity, and frequency attributes of symptoms by level. Responses are detailed with regard to percentage of time each symptom is seen at a given level. Most issues reported in these 2 categories were moderate or mild, with the exception of hair loss frequency. PRO-CTCAE = Patient-Reported Outcomes version of the Common Terminology Criteria for Adverse Events.

**Figure 4.**
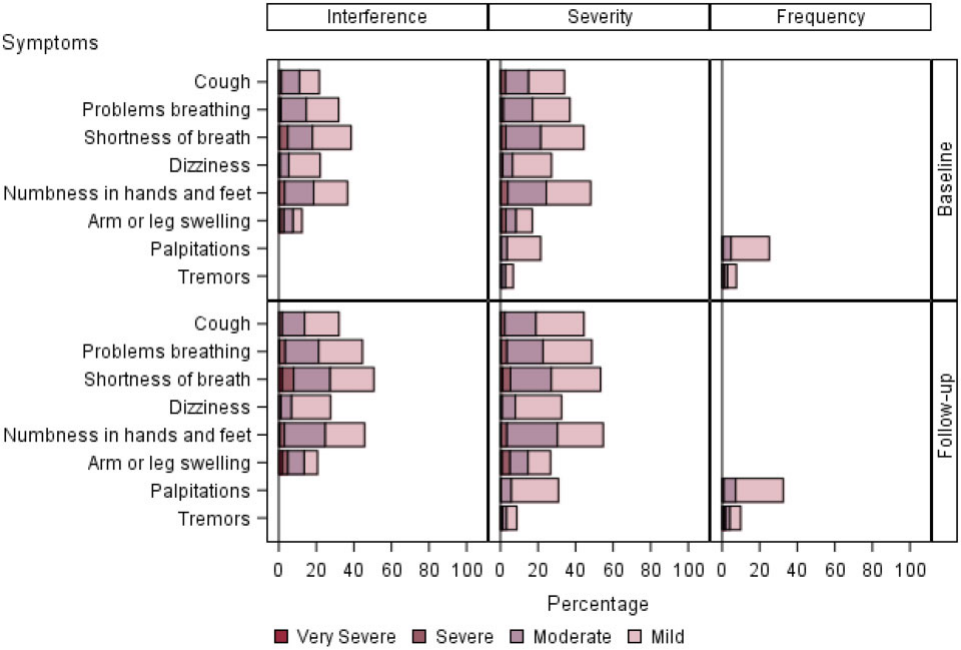
PRO-CTCAE symptoms rated in the respiratory, neurologic, and cardiovascular. Bar chart panels highlight symptom attribute and level over time. Bar chart panels break down interference, severity, and frequency attributes of symptoms by level. Responses are detailed with regard to percentage of time each symptom is seen at a given level. Most patient-reported respiratory symptoms were mild or moderate, with some increase in incidence seen from baseline to follow-up. PRO-CTCAE = Patient-Reported Outcomes version of the Common Terminology Criteria for Adverse Events.

Details of PRO-CTCAE symptom attribute and level over time are displayed in Figures 1-4. We separated symptom attributes into the categories of mood, pain, sleep, and attention (Figure 1); gastrointestinal (Figure 2); cutaneous and oral (Figure 3); and respiratory, neurologic, and cardiovascular (Figure 4). Low-frequency items (sexual, gynecologic and/or urinary, other miscellaneous) were not included in the graphs.

Mood, pain, sleep, and attention were common patient-reported issues (Figure 1). Although most symptoms were reported as either mild or moderate in severity, fatigue and lack of energy were more commonly severe or very severe (16.2%). Pain was commonly reported as severe (6.3%) and interfering “quite a bit” or “very much” (7.0%).

Gastrointestinal adverse events (Figure 2) were reported with higher severity. Appetite was one of the most common issues reported (47.0%) and increased in severity at follow-up (6.3% severe or very severe at baseline to 9.2% at follow-up). Cutaneous and oral symptoms (Figure 3) were largely mild or moderate, with the exception of frequency of hair loss (13.4% at baseline). Interference with skin problems (baseline 12.9% to 18.1% follow-up) and dry-mouth severity (2.3% baseline to 3.1% follow-up) were more commonly reported between baseline and follow-up.

Respiratory, neurologic, and cardiovascular symptoms were mostly mild or moderate (Figure 4). However, the severity of cough, dyspnea, dizziness, numbness, swelling, and tremors had increased.

Results related to correlation of derived aggregate scores at baseline for frequency, severity, and interference PRO-CTCAE items to previously validated tools are displayed in Table 4. (Correlation coefficients using scores at subsequent time points [week 4 and week 12] were also computed, yielded comparable results, and were not included.) Overall, PRO-CTCAE was positively correlated with distress scale and negatively correlated with FACT-G. The correlation coefficient (*r*) for psychologic distress scale was low (frequency: Pearson *r *=* *0.33; severity, *r *=* *0.43; and symptom interference r = 0.46). For FACT-G (total scores), correlation with PRO-CTCAE symptom frequency (Pearson *r* = -0.45), severity (Pearson *r* = -0.67), and symptom interference (Pearson *r* = -0.69) was moderate.

**Table 4.** Pearson correlation coefficient (ρ) for distress level, FACT-G (total and subscale scores), and PRO-CTCAE items recorded at baseline^a^

Questionnaire	PRO-CTCAE symptom frequency	PRO-CTCAE symptom severity	PRO-CTCAE symptom interference
All PRO-CTCAE items^b^			
Distress level	0.33	0.43	0.46
Fact-G index	−0.45	−0.67	−0.69
PWB subscore	−0.48	−0.72	−0.67
SWB subscore	−0.19	−0.27	−0.27
EWB subscore	−0.18	−0.32	−0.42
FWB subscore	−0.45	−0.63	−0.63
PRO-CTCAE symptom frequency	—	0.71	0.61
PRO-CTCAE symptom severity	—	—	0.86
Mood items only (anxiety and depression)^c^			
Distress level	0.57	0.60	0.55
Fact-G index	−0.54	−0.60	−0.57
PWB subscore	−0.33	−0.39	−0.36
SWB subscore	−0.22	−0.26	−0.24
EWB subscore	−0.63	−0.64	−0.64
FWB subscore	−0.41	−0.47	−0.44
PRO-CTCAE symptom frequency	—	0.83	0.76
PRO-CTCAE symptom severity	—	—	0.88

a All results highly statistically significant, *P* < .0001. Em dash indicates item is correlated with itself, or correlation of 2 items is repeated in the transpose position. EWB = emotional well-being; FWB = functional well-being; PRO-CTCAE = Patient-Reported Outcomes version of the Common Terminology Criteria for Adverse Events; PWB = physical well-being; SWB = social/family well-being.

b All PRO-CTCAE items, summarized over all PRO-CTC items with frequency (n = 10), severity (n = 39), or interference (n = 21) attributes, respectively.

c Mood items only (anxiety and depression), summarized overall mood-related PRO-CTC items with frequency (n = 1), severity (n = 2), or interference (n = 2) attributes.

The moderate correlation between the PRO-CTCAE tool and the physical well-being subscale of FACT-G (Table 4) provides evidence of concurrent validity, because both measures explore related constructs in symptom assessment. Similar results were noted for PRO-CTCAE symptom interference (Table 4). Symptom frequency had lower correlations with all attributed items, with Pearson correlation coefficient ranging from 0.18 to 0.48.

Table 4 focuses on PRO category of mood items of the PRO-CTCAE (anxiety and depression). Stronger Pearson correlation coefficients were noted between distress level and all PRO-CTCAE mood attributes: symptom frequency (Pearson *r* = 0.57), symptom severity (Pearson *r* = 0.60), and symptom interference (Pearson *r* = 0.55). Similar correlations were also noted between PRO-CTCAE mood-related items and FACT-G total score (Pearson *r* = -0.54 to -0.6), with strongest correlation among the EWB subscore. Overall, symptom severity explained a larger proportion of variability in distress and FACT-G than interference or frequency. Correlations between PRO-CTCAE items and corresponding FACT-G (total and subscales) and distress levels reached statistical significance for all items (*P* <.001).

## Discussion

This secondary analysis provides evidence supporting the added value of PRO-CTCAE to measure the symptoms of patients enrolled onto phase I oncology trials. We noted small to moderate correlations for distress level for all PRO-CTCAE items (Pearson *r *=* *0.33-0.46) and moderate correlations with QOL as measured by FACT-G total (Pearson *r* = −0.45 to −0.69).

Strengths of this study include a diverse patient sample with respect to age and disease site, with enrichment of less common cancers (pancreatic, kidney, sarcoma). Both institutions involved in the study are leaders in phase I therapeutics. We focused on patients treated on phase I trials for advanced cancer given a high frequency of symptomatic adverse events. As patients accrue new toxicities or worsening of baseline symptoms over the course of treatment, it is anticipated to observe a change in QOL or distress levels. In addition, 30% of participants were of minority population, reflecting the feasibility of survey administration to a range of racial backgrounds.

Our primary objective was to investigate the association of symptomatic toxicities, as measured by PRO-CTCAE, with global quality of life and psychological distress anchors. Demonstrating a correlation between symptoms, patient quality of life, and distress are important for several reasons. First, the US Food and Drug Administration has identified symptomatic AEs, physical function, and patient QOL as priority areas of interest for PRO analysis (41). Second, although phase I trials are primarily focused on dose finding and a preliminary assessment of the safety of a new agent or drug combination, several investigators have suggested expanding the definition of a dose-limiting toxicity to include PRO data (42). A deeper understanding of how symptoms impact patient QOL and distress informs on the overall tolerability of a cancer therapeutic. As it now stands, lower-grade toxicities below the threshold of the drug-limiting toxicity definition elude current methods for AE analysis and may underestimate drug contribution to patient well-being (43). Therefore, establishing correlation between patient-reported toxicities as measured by PRO-CTCAE and QOL has potential to capture the impact of cumulative lower-grade toxicities.

Recent studies have demonstrated underreporting by physicians, compared with patients, on common symptoms of anorexia, nausea, constipation, diarrhea, and hair loss (44). In this study, many symptoms, such as bloating of the abdomen, constipation, problems with memory and concentration, frequent urination, dry mouth, anxiety, depression, and shortness of breath, affected nearly 40% of patients and were rated as severe or very severe (Table 3). These symptoms would be missed using global health-related QOL assessment tools (FACT-G). Our data also demonstrates that symptoms are experienced differently by patients, with distinct quality, frequency, intensity, and levels of interference. For example, hiccups (19%) and easy bruising (22%) were frequent problems but were almost never identified as severe or interfering with daily activities, whereas urination not only occurred frequently (39.9%) but also interfered with activities (26.1%) and was noted as a high-grade toxicity (scored ≥ 3) by 9% of patients. Therefore, it is important to measure not only the presence of a symptom but also the distinct symptom experience and how it impacts patient-reported overall quality of life. This heightened level of awareness would allow clinicians to better target the psychosocial needs of patients.

Correlating PRO-CTCAE and distress level is similarly important, because patients with advanced cancer often experience distress associated with disease-related symptoms or treatment-related side effects. In a preliminary study of the trial reported here, emotional distress levels for patients were high (45). The average overall distress on the Distress Thermometer was 3.6, with scores above 3 generally requiring clinical assessment and intervention (46). Stronger associations were noted for mood items of the PRO-CTCAE only with distress level (*r *=* *0.55-0.6), and PRO-CTCAE symptom interference scores had the strongest correlation with distress level (Pearson *r *=* *0.46) and FACT-G total (Pearson *r* = -0.69). Previous investigators have documented the negative relationship between symptom distress and QOL, both physically and emotionally (47,48).

The PRO-CTCAE was not intended to combine individual items; the best way to combine the attributes (frequency, severity, interference) and how to interpret the scores has not been established and is under study. Dueck and colleagues recently presented a novel scoring algorithm for mapping PRO-CTCAE individual item scores into a single composite AE grade (39). Our intent of formulating an aggregate score was to explore whether symptom clusters in subcategories (interference, frequency, severity) would better characterize the patient experience. This is consistent with guidance from the NCI recommending descriptive reporting of available attribute (49). Importantly, our work enhances the interpretability and utility of PRO-CTCAE and adds to the currently sparse literature.

Several caveats and limitations should be considered. Our study was conducted in an English-speaking, US-residing patient population and limited in this regard. Second, we assessed convergent validity, but other measures of construct validity, such as divergent, discriminative, and predictive validity, are warranted. Third, the items tested were correlated with FACT-G and distress anchors, both of which were not widely used in validation and reliability studies to date. Future work will be critical regarding which modifications could be made to existing HRQOL instruments to reduce duplication and patient burden, with the ultimate goal of achieving a comprehensive evaluation of the patient experience most affected by therapy while maximizing the relevance of individual questions and minimizing duplicative work.

In conclusion, the results of this study suggest PRO-CTCAE is correlated with validated patient-reported tools measuring general quality of life and psychological distress and can achieve its intended aim to amplify the patient’s voice. Further validation and additional psychometric work is needed to advance the clinical utility of PROs.

## Funding

This research is supported by a research grant from NCI-RO1 CA177562, “Integration of Palliative Care for Cancer Patients on Phase 1 Trials” (B. Ferrell, T. Smith: Co-PIs); the City of Hope Core, NCI P30CA033572; and the Johns Hopkins Sidney Kimmel Comprehensive Cancer Center Core Grant, NCI.

## Notes

**Role of the funders:** The funders had no role in the design of the study; the collection, analysis, and interpretation of the data; the writing of the manuscript; and the decision to submit the manuscript for publication.

**Conflicts of interest**: The authors have no conflicts of interest to report.

**Role of the authors:** All authors contributed to review and revision. BF and TJS wrote grant applications. All authors performed data analysis and interpretation. RS, BF, NR, TJS drafted the manuscript. All authors contributed to editing and critical revision for important intellectual content.

**Disclaimer:** The content is solely the responsibility of the authors and does not necessarily represent the official views of the National Institutes of Health.

## Data Availability Statement

Not applicable.
